# 47. Activated PI3 kinase delta syndrome: a rare cause of inflammatory disease

**DOI:** 10.1093/rap/rky034.010

**Published:** 2018-09-20

**Authors:** Nehal Narayan, Peter Hewins, Peter Lane, Benjamin Rhodes

**Affiliations:** 1 Rheumatology, University Hospitals Birmingham, Birmingham, UNITED KINGDOM; 2 Nephrology, University Hospitals Birmingham, Birmingham, UNITED KINGDOM; 3 Immunology, University Hospitals Birmingham, Birmingham, UNITED KINGDOM

**Introduction:** Activated PI3 kinase delta syndrome (APDS) is a recently identified rare, but clinically severe primary immunodeficiency, resulting from heterozygous gain of function mutations that induce hyperactivation of class I phosphoinositide 3 kinase delta (PI3Kδ). PI3Kδ is well recognised to be a critical component of pro-inflammatory signalling pathways in leukocytes, predominantly lymphocytes. Certainly, PI3Kδ has long been implicated in the pathogenesis of inflammatory diseases such as rheumatoid arthritis. Patients with APDS have features of both primary immunodeficiency, such as recurrent chest infections, viral warts, but also widespread lymphadenopathy, lymphoma and autoimmune inflammatory disease, such as arthritis and glomerulonephritis. Indeed, inflammatory disease has been reported in over a third of patients in cohort studies of APDS. Here, we describe a patient presenting to our rheumatology department with classic features of APDS. We review the common clinical manifestations of this rare, but clinically severe disease, and summarise current and future management strategies.

**Case description:** Our patient is a 39 year old male. He presented to hospital with several days of sweats, a cough productive of green sputum, and an episode of two cup-fulls of frank haemoptysis. His past medical history included recurrent chest infections since the age of six months, recurrent middle ear infections, lifelong asthma, and a persistent IgG and IgM monoclonal gammopathy with a persistent borderline leucopenia and thrombocytopenia, under surveillance by a haematologist. He described a five year history of recurrent viral warts, affecting his hands and feet. On examination, he was afebrile and not in respiratory distress. Auscultation of his chest revealed bibasal respiratory crackles, and a chest x-ray confirmed consolidation and possible bronchiectatic change. Sputum grew mixed species; the gram negative enterobacteriaceae *Raoultella sp. and Kluyvera sp.* as well as *Pseudomonas fluorescens.* He was treated with antibiotics for a chest infection, and his symptoms improved. A CT thorax confirmed bronchiectasis, with evidence of pulmonary nodules, hepatosplenomegaly, liver nodules and scattered mediastinal lymphadenopathy. Urine dip demonstrated +++ protein, and albumin: creatinine ratio was 301 mg/ml, although eGFR remained normal. A renal biopsy demonstrated signs consistent with lupus nephritis (glomerular sclerosis, glomerular membrane thickening, interstitial inflammation, with positive mesangial staining for IgG, IgA, C3d and C1q). Biopsy of a liver nodule confirmed marginal zone lymphoma, and histology of a supraclavicular lymph node demonstrated paracortical histiocytes, and melanin-laden macrophages, consistent with a dermopathic lymphadenopathy, a known association of lymphoma. The patient underwent genotyping for PI3Kδ mutations, and the laboratory confirmed a gain of function mutation in *PI3KCD,* the catalytic subunit of PI3Kδ. He was scheduled to enter into a clinical trial aiming to utilise a PI3Kδ inhibitor to control his disease, and his disease remains stable.

**Discussion:** APDS was first classified in 2013, where cohorts of patients with primary immunodeficiencies of unknown cause underwent whole-exome sequencing. This identified a rare heterozygous gain-of-function mutation in *PI3KCD,* the catalytic subunit of PI3Kδ. Subsequently, further mutations in *PI3KR1,* the p85α subunit of PI3Kδ, were described. APDS resulting from mutations in *PI3KCD* were classified as APDS1 and disease resulting from mutations in *PI3KR1* were classified as APDS 2. Several cohort studies describe the salient clinical features of APDS1 and 2, and the prevalence of main clinical features (see Table 1). Management of APDS includes antibiotic prophylaxis and immunoglobulin replacement, to reduce the incidence of infection. Immunosuppressive therapies are targeted to reduce proliferation and activation of lymphocytes; previous successful treatment has been reported with rituximab, azathioprine and also rapamycin. Rapamycin inhibits the downstream PI3K effector mTOR pathway, and has been demonstrated in a cohort study of APDS to be effective for the management of lymphoproliferative disease in APDS. Even more specific to the gain-of-function mutations of PI3Kδ in APDS, are the newly developed PI3Kδ inhibitors. The PI3Kδ inhibitor idealisib is already license for use in chronic lymphocytic leukaemia and non-Hodgkin lymphoma. However, utility of this drug is often limited due to its considerable side effect profile, including pneumonitis, and colitis, thought to be related to total suppression of the actions of PI3Kδ. In contrast, a recent pilot trial of the PI3Kδ inhibitor leniolisib in 6 patients with APDS has demonstrated the drug to be well tolerated, and able to ameliorate lymphoproliferation, with significant reduction in lymph node and spleen size by twelve weeks. The efficacy of PI3Kδ inhibitors to ameliorate inflammatory disease outside lymph nodes in patients with APDS may lend further insight into the potential for PI3Kδ inhibitors to be used as treatment for inflammatory diseases such as rheumatoid arthritis, where PI3Kδ is known to have an important role in disease pathogenesis.

**Key Learning Points:** APDS is a rare disease with potential serious complications, presenting with symptoms of immunosuppression and inflammatory disease. The disease can be diagnosed by genotyping for PI3Kδ mutations. Further data on the success of treatment of APDS using targeted PI3Kδ inhibitors may yield further insight into potential utility of PI3Kδ inhibitors for the treatment of inflammatory diseases, including RA, where PI3Kδ is already known to play an important role in disease pathogenesis.

**Table:** Table 1: Reported prevalence of clinical features of APDS (adapted from Michalovich and Nejentsev 2018)

Manifestations	APDS 1	APDS 2
Recurrent respiratory tract infections	96%	100%
bronchiectasis	60%	18%
Herpesvirus infections	49%	31%
lymphadenopathy	64%	75%
splenomegaly	58%	43%
autoimmune or autoinflammatory disease	34%	17%
lymphoma	13%	25%

**Disclosure: N. Narayan:** None. **P. Hewins:** None. **P. Lane:** None. **B. Rhodes:** None.

